# Biologics and Small‐Molecule Therapies in Netherton Syndrome: A Comprehensive Review

**DOI:** 10.1111/1346-8138.17923

**Published:** 2025-09-01

**Authors:** Shin Morizane, Tomoyuki Mukai, Ko Sunagawa, Ken‐ichi Hasui, Anri Morita, Hayato Nomura, Mamoru Ouchida

**Affiliations:** ^1^ Department of Dermatology Okayama University Graduate School of Medicine, Dentistry and Pharmaceutical Sciences Okayama Japan; ^2^ Department of Immunology and Molecular Genetics Kawasaki Medical School Kurashiki Japan; ^3^ Department of Molecular Oncology Okayama University Graduate School of Medicine, Dentistry and Pharmaceutical Sciences Okayama Japan

## Abstract

Netherton syndrome (NS) is a rare congenital ichthyosis caused by loss‐of‐function mutations in the SPINK5 gene, leading to defective expression of the serine protease inhibitor LEKTI. Dysregulated epidermal protease activity results in impaired skin barrier function and chronic inflammation, accompanied by complex immune profiles. NS patients commonly show activation of the inflammatory axis, centered on IL‐17 and IL‐36, in the skin and blood, and show a psoriasis‐like shift to Th17. Conversely, the immune profile differs depending on the clinical type, with ichthyosis linearis circumflexa type characterized by complement activation and Th2‐type allergic responses, and scaly erythroderma type characterized by a type I IFN signature and Th9‐type allergic responses. While symptomatic treatments such as emollients and topical corticosteroids have been the mainstay of care, recent advances have opened new therapeutic avenues involving biologic agents and oral small‐molecule immunomodulators. This review provides a comprehensive overview of the current clinical landscape and future directions of biologics (e.g., dupilumab, secukinumab, ustekinumab) and small‐molecule therapies (e.g., JAK inhibitors such as tofacitinib, baricitinib, and upadacitinib) in the treatment of NS. Though evidence remains limited to case reports and small series, preliminary data suggest that cytokine‐targeted interventions—particularly those inhibiting IL‐4, IL‐13, IL‐17, IL‐36, and JAK pathways—may offer tangible clinical benefits. Well‐designed clinical trials and mechanistic investigations are crucial to establishing their place in NS management.

**Abbreviations**
(KLKs): kallikrein‐related peptidases
(LEKTI): lymphoepithelial Kazal‐type‐related inhibitor
(NS): Netherton syndrome
(PAR2): protease‐activated receptor 2

## 1 Introduction

The stratum corneum is the outermost layer of the body surface, and protects against external stimuli while also contributing to moisture retention within the skin. The stratum corneum peels off to remove foreign bodies, impurities, and pathogens from the skin surface. One of the important factors in this peeling is the serine protease kallikrein‐related peptidases (KLKs), and 15 types of KLKs have been reported in humans [1]. In particular, KLK5 (trypsin‐type serine protease) and KLK7 (chymotrypsin‐type serine protease) are highly expressed in epidermal keratinocytes [2, 3, 4]. These KLKs promote the desquamation of the stratum corneum by cleaving cell adhesion molecules such as desmoglein 1, desmocollin 1, and corneodesmosin between epidermal keratinocytes [5]. While epidermal keratinocytes express these KLKs, they also express serine protease inhibitors, which strictly regulate the activity of KLKs to prevent excessive activity [6, 7, 8].

Lympho‐epithelial Kazal‐type‐related inhibitor (LEKTI) is a major serine protease inhibitor protein produced by epidermal keratinocytes, and the importance of LEKTI has been found in patients with Netherton syndrome (NS) [9]. NS is a rare autosomal recessive genodermatosis, and the exact prevalence remains uncertain, but estimates suggest an incidence of less than 1 in 200 000 live births [10]. In NS patients, loss‐of‐function mutations in the SPINK5 gene encoding LEKTI result in a decrease or disappearance of LEKTI inhibitor activity, leading to the inability to suppress stratum corneum serine protease activity, resulting in excessive peeling of the stratum corneum and the appearance of atopic dermatitis (AD)‐like symptoms [9]. In addition to the triad of ichthyosis, hair abnormalities, and atopic predisposition, it is known that patients with NS are also prone to complications such as low body weight, short stature, growth retardation, and generalized aminoaciduria [9]. The degree of loss of inhibitor activity varies depending on the location of the mutation in the SPINK5 gene, and the effect on the protease activity of the epidermal KLK group also differs. In cases where the mutation occurs closer to the 5′ end of the SPINK5 gene, protease activity is hardly suppressed, and it is thought to be a more severe form [9]. In cases where a homozygote mutation occurs in domain 1 of LEKTI, the entire inhibitor domain is lost, and it has been reported that patients die early after birth due to skin barrier disorders such as severe dehydration [11].

The definitive treatment for NS would be to supplement with LEKTI or administer a protease inhibitor with similar activity, but there are currently no approved drugs. Until a few years ago, the only treatments available were symptomatic, such as moisturizers like petrolatum or ointments containing mild steroids to suppress inflammation and itching; there has been a strong demand for the development of new therapeutic drugs based on the mechanism of disease.

**FIGURE 1:**
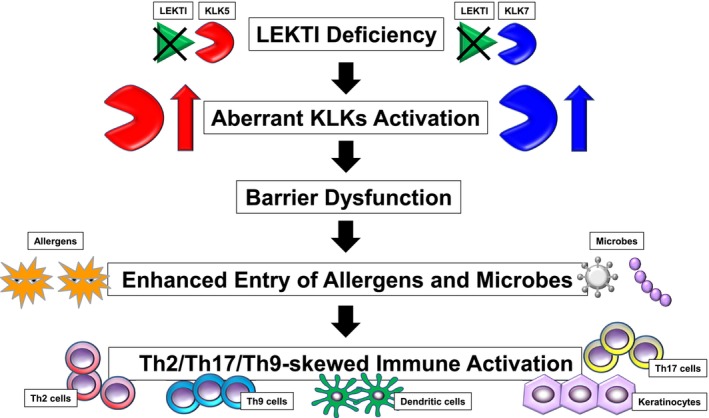
Pathophysiological mechanism of skin barrier dysfunction and inflammatory cascade in LEKTI deficiency. In LEKTI‐deficient skin, aberrant activation of epidermal kallikreins (KLK5 and KLK7) leads to impaired skin barrier function. This dysfunction facilitates the enhanced entry of allergens and microbes through the compromised stratum corneum. The resulting exposure triggers complex immune responses, involving keratinocytes, dendritic cells, and T helper cell subsets including Th2, Th17, and Th9. These interactions drive chronic inflammation and contribute to the clinical manifestations observed in LEKTI‐deficient conditions such as Netherton syndrome.

Deficiency of LEKTI leads to abnormal activation of KLKs, which disrupts the skin barrier function and allows allergens and microorganisms to more easily penetrate the epidermis, triggering complex immune responses and inflammation (Figure 1) [12]. However, recent advances in multi‐omics analysis have improved our understanding of the immune profile of NS. NS patients commonly show activation of the inflammatory axis, centered on IL‐17 and IL‐36, in the skin and blood, and show a psoriasis‐like shift to Th17 [13]. On the other hand, the immune profile differs depending on the clinical type, with NS‐ichthyosis linearis circumflexa (ILC) type characterized by complement activation and Th2‐type allergic responses (increased IL‐4, IL‐13, and CCL27), and NS‐scaly erythroderma (SE) type characterized by a type I IFN signature and Th9‐type allergic responses (increased CCL17/TARC and CCL22/MDC) [13]. Such complex inflammation is thought to be triggered by the invasion of allergens and microbes (Figure 1). In addition to Th2, Th9, and Th17 cells, other types of cells including keratinocytes and dendritic cells are thought to be deeply involved in both pathological conditions (Figure 1).

Moreover, in recent years, there have been an increasing number of case reports in which patients with NS have been administered biologics or small‐molecule therapies, and their symptoms have improved (Table 1). However, there is controversy as to which preparation provides the best improvement and whether different preparations should be used depending on the case. This review describes the current status and future prospects of these treatments for NS.

**TABLE 1:** Biologic and small‐molecule therapies reported for Netherton syndrome: mechanisms, clinical outcomes, and references.

Agent name	Target/mechanism	Reported cases	Outcome summary	References
Dupilumab	IL‐4Rα	42	Mostly effective; improves erythema, pruritus, and barrier function	[14, 15, 16, 17, 18, 19, 20, 21, 22, 23, 24, 25, 26, 27, 28, 29, 30, 31, 32, 33, 34, 35, 36, 37, 38, 39, 40, 41, 42]
Secukinumab	IL‐17A	27	Effective in both ILC and SE types; some combined with dupilumab	[27, 30, 32, 38, 43, 44, 45, 46, 47, 48, 49, 50]
Ixekizumab	IL‐17A	7	Effective; overlaps with secukinumab cases	[14, 37, 51, 52]
Ustekinumab	IL‐12/23 (p40)	6	Mixed results	[37, 51, 53, 54]
Spesolimab	IL‐36R	1	Marked improvement in a pediatric case	[55, 56]
Infliximab	TNF‐α	5	Partially effective; reduces erythroderma/pruritus	[57, 58, 59, 60]
Adalimumab	TNF‐α	2	Partial/transient effect	[43, 44]
Anakinra	IL‐1R antagonist	4	Limited efficacy	[14]
Omalizumab	IgE	6	Mostly ineffective	[27, 43, 49, 61, 62]
Others (e.g., Tralokinumab, Lebrikizumab, Mepolizumab, Tocilizumab, Brodalumab, Bimekizumab, Nemolizumab, Anifrolumab)	Various (IL‐13, IL‐5, IL‐6R, IL‐17RA, IL‐17A/F, IL‐31RA, IFNAR1)	None	No reports yet in NS	—
Tofacitinib	JAK1/3	1	Effective in 3‐year‐old boy	[63]
Baricitinib	JAK1/2	1	Effective in dupilumab‐resistant SE‐NS	[31]
Upadacitinib	JAK1	4	Variable; some marked improvement	[30, 33, 64, 65]
Abrocitinib	JAK1	2	Effective and well‐tolerated	[38, 66]
Ritlecitinib	JAK3 + TEC	None	No reports in NS	—
Deucravacitinib	TYK2	None	No reports in NS	—
Apremilast	PDE4	None	No reports in NS	—
Infliximab	TNF‐α	5	Partially effective; reduces erythroderma/pruritus	[59, 60, 67, 68]
Adalimumab	TNF‐α	2	Partial/transient effect	[69, 70]
Anakinra	IL‐1R antagonist	4	Limited efficacy	[71]
Dupilumab	IL‐4Rα	43	Mostly effective; improves erythema, pruritus, and barrier function	[17, 18, 19, 20, 21, 22, 23, 24, 25, 26, 27, 28, 29, 30, 31, 32, 33, 34, 35, 36, 37, 38, 39, 40, 41, 42, 71, 72, 73, 74]
Ustekinumab	IL‐12/23 (p40)	6	Mixed results	[39, 45, 75, 76]
Secukinumab	IL‐17A	27	Effective in both ILC and SE types; some combined with dupilumab	[29, 32, 34, 40, 48, 49, 50, 52, 69, 70, 77, 78]
Ixekizumab	IL‐17A	7	Effective; overlaps with secukinumab cases	[39, 45, 71, 79]
Spesolimab	IL‐36R	1	Marked improvement in a pediatric case	[80]
Omalizumab	IgE	6	Mostly ineffective	[29, 52, 69, 81, 82]
Others (e.g., Mepolizumab, Tocilizumab, Tralokinumab, Lebrikizumab, Brodalumab, Bimekizumab, Guselkumab, Risankizumab, Tildrakizumab, Nemolizumab, Anifrolumab, Tezepelumab)	Various (IL‐5, IL‐6R, IL‐13, IL‐17RA, IL‐17A/F, IL‐23p19, IL‐31RA, IFNAR1, TSLP)	None	No reports in NS	—
Tofacitinib	JAK1/3	1	Effective in 3‐year‐old boy	[65]
Baricitinib	JAK1/2	1	Effective in dupilumab‐resistant SE‐NS	[33]
Upadacitinib	JAK1	4	Variable; some marked improvement	[32, 35, 83, 84]
Abrocitinib	JAK1	2	Effective and well‐tolerated	[40, 85]
Ritlecitinib	JAK3 + TEC	None	No reports in NS	—
Deucravacitinib	TYK2	None	No reports in NS	—
Apremilast	PDE4	None	No reports in NS	—

*Abbreviations:* ILC, ichthyosis linearis circumflexa; NS, Netherton Syndrome; SE, scaly erythroderma.

## 2 Biologics in Netherton Syndrome

### 2.1 TNF Inhibitors

#### 2.1.1 Infliximab

Infliximab is a chimeric human‐murine monoclonal antibody against TNF‐α [57]. It has been approved for the treatment of psoriatic arthritis (PsA), plaque psoriasis (PP), rheumatoid arthritis (RA), ankylosing spondylitis (AS), Crohn's disease (CD), ulcerative colitis (UC), and juvenile idiopathic arthritis (JIA) (all indications in this review are based on approvals by the US Food and Drug Administration or equivalent global regulatory authorities, unless otherwise specified) [58]. Case reports describe clinical improvement in NS patients, particularly in reducing erythroderma and pruritus, although responses are variable [59, 60, 67, 68].

#### 2.1.2 Adalimumab

Adalimumab is a fully human IgG1 monoclonal antibody that neutralizes both soluble and membrane‐bound TNF‐α [43]. Approved indications include RA, AS, PsA, PP, hidradenitis suppurativa (HS), JIA, CD, UC, and uveitis [44]. Adalimumab has shown partial and transient benefit in 2 cases with NS [69, 70].

#### 2.1.3 Certolizumab Pegol

Certolizumab pegol is a PEG‐conjugated Fab' fragment against TNF‐α that lacks Fc and is approved for the treatment of CD, RA, AS, nonradiographic axial spondyloarthritis (nr‐axSpA), PP, and PsA [86]. To date, there are no published reports on the use of this agent in NS.

### 2.2 IL‐1 Inhibitors

#### 2.2.1 Anakinra

Anakinra is a human IL‐1 receptor antagonist protein and acts on both IL‐1α and IL‐1β [14]. It is approved for the treatment of RA, systemic juvenile idiopathic arthritis (SJIA), cryopyrin‐associated periodic syndrome (CAPS), and so forth [87]. Four cases with NS have been reported to be treated with Anakinra, but they were less effective [71].

#### 2.2.2 Canakinumab and Rilonacept

Canakinumab is a human monoclonal antibody that targets interleukin‐1β and is approved for the treatment of CAPS, Tumor Necrosis Factor Receptor Associated Periodic Syndrome (TRAPS), Hyperimmunoglobulin D Syndrome (HIDS)/Mevalonate Kinase Deficiency (MKD), Familial Mediterranean Fever (FMF), and Active Systemic SJIA [14, 88]. Rilonacept is an IL‐1 receptor fusion protein consisting of the Fc portion of human IgG1 and the human IL‐1 receptor, which blocks both IL‐1A and IL‐1β and is approved for the treatment of CAPS, Deficiency of Interleukin‐1 Receptor Antagonist (DIRA), and recurrent pericarditis (RP) [14, 89]. There are no reports in NS to date.

### 2.3 IL‐4/IL‐13 and IL‐13 Inhibitors

#### 2.3.1 Dupilumab

Dupilumab is a human monoclonal antibody against the IL‐4 receptor alpha subunit and blocks IL‐4 and IL‐13 signals [15]. It is approved for the treatment of AD, asthma, chronic rhinosinusitis with nasal polyposis, eosinophilic esophagitis, and prurigo nodularis (PN), and chronic idiopathic/spontaneous urticaria (CSU) [15, 16]. NS‐ILC type shows elevated blood levels of TH2 cytokines (IL‐4, IL‐13) and CCL27 and has an immune background similar to that of AD; therefore, dupilumab, which suppresses the TH2 axis, is theoretically thought to be effective [13]. It is the most widely reported biologic agent in NS (more than 40 cases), with many cases showing significant improvement in erythema, pruritus, and skin barrier integrity, although some have shown no benefit [17, 18, 19, 20, 21, 22, 23, 24, 25, 26, 27, 28, 29, 30, 31, 32, 33, 34, 35, 36, 37, 38, 39, 40, 41, 42, 71, 72, 73, 74]. Especially, dupilumab has consistently demonstrated strong clinical benefit across several pediatric NS cases—ranging from infants to older children—including rapid relief of pruritus, reduction in scaling/erythroderma, and improved growth or quality of life [19, 26, 27, 30, 31, 74]. Among them, a 6‐year‐old boy maintained disease control over 3 years of continuous treatment without major adverse events [74]. A double‐blind randomized placebo‐controlled study evaluating the efficacy and safety of dupilumab for the treatment of NS is ongoing (NCT04244006) [90].

#### 2.3.2 Tralokinumab and Lebrikizumab

Both tralokinumab and lebrikizumab are monoclonal antibodies targeting interleukin‐13 (IL‐13), a key cytokine in the TH2‐mediated immune response. To date, there have been no reports of these uses in NS; though their mechanism aligns with the Th2 profile of many NS patients.

### 2.4 IL‐5 Inhibitors

#### 2.4.1 Mepolizumab, Reslizumab, Benralizumab

Mepolizumab and reslizumab are humanized anti‐IL‐5 antibodies, and benralizumab is a humanized IL‐5 receptor alpha‐effective cytolytic monoclonal antibody [91]. There have been no reports of these uses in NS to date. Eosinophilic involvement is not consistently dominant in NS, possibly limiting their utility.

### 2.5 IL‐6 Inhibitors

#### 2.5.1 Tocilizumab and Sarilumab

Both tocilizumab and sarilumab are monoclonal antibodies targeting IL‐6 receptor and blocking IL‐6 signaling [53, 54]. There have been no reports of these uses in NS to date.

### 2.6 IL‐12/IL‐23 and IL‐23 Inhibitors

#### 2.6.1 Ustekinumab

Ustekinumab is a monoclonal antibody against the p40 subunit common to both IL‐12 and IL‐23 [51]. Although the uses of ustekinumab have been reported in adolescents and adults with NS, the effectiveness of the treatment varies widely [39, 45, 75, 76].

#### 2.6.2 Guselkumab, Risankizumab, Tildrakizumab

Guselkumab, risankizumab, and tildrakizumab are monoclonal antibodies against the p19 subunit of IL‐23 [46]. There have been no reports of these uses in NS to date.

### 2.7 IL‐17 Inhibitors

#### 2.7.1 Secukinumab

Secukinumab is a human monoclonal antibody that selectively targets IL‐17A and is approved for the treatment of PP, PsA, AS, nr‐axSpA, and pediatric PP [47]. The IL‐17/IL‐36 signaling axis has been identified as a key pathogenic pathway in both scaly erythroderma‐type and ichthyosis linearis circumflexa‐type NS. Notably, the clinical efficacy of secukinumab has been reported by several independent groups [29, 32, 34, 40, 48, 49, 50, 52, 69, 70, 77, 78]. Interestingly, the combination therapy of dupilumab and secukinumab has been shown to be quite effective [34]. This prospective pilot study is the first to assess combined secukinumab and dupilumab in pediatric Netherton syndrome, targeting both Th2 and Th17 inflammation. Strengths include novelty, a prospective design, multiple validated endpoints, supportive biomarker profiling, and a favorable short‐term safety profile. However, limitations include the very small sample size (*n* = 7), lack of a control group, short follow‐up (24 weeks), possible bias from LOCF data imputation, one discontinuation due to cost, and the single‐center setting in China. While results suggest rapid, marked improvement, confirmatory multicenter controlled trials with longer follow‐up are needed to establish efficacy, safety, and real‐world applicability.

#### 2.7.2 Ixekizumab

Ixekizumab is a humanized monoclonal antibody that selectively targets IL‐17A and is approved for the treatment of PP, PsA, AS, nr‐axSpA, and pediatric PP [92]. Similar to secukinumab, the efficacy of ixekizumab has been reported by several groups [39, 45, 71, 79].

#### 2.7.3 Brodalumab

Brodalumab is a human monoclonal antibody against IL‐17 receptor A and neutralizes the effect of IL‐17A, IL‐17C, IL‐17E, IL‐17F, and heterodimeric IL‐17A/F [93]. To date, there have been no reports of the use in NS, although the IL‐17/IL‐36 axis predominance has been shown in NS patients [13].

#### 2.7.4 Bimekizumab

Bimekizumab is a human monoclonal antibody against IL17A, IL‐17F, and IL‐17A/F [55]. Same as brodalumab, there have been no reports of its use in NS to date; although the IL‐17/IL‐36 axis predominance has been shown in NS patients.

### 2.8 IL‐31 Inhibitor

#### 2.8.1 Nemolizumab

Nemolizumab is a humanized monoclonal antibody against IL‐31 receptor A and is approved for the treatment of pruritus of AD and PN [56]. Considering that IL‐31 plays a central role in pruritus and is also clinically important in diseases such as AD and PN, it is suggested that IL‐31 may play an important role in the pathogenesis of NS. However, there have been no reports to date on the relationship between NS and IL‐31 or nemolizumab.

### 2.9 IL‐36 Inhibitors

#### 2.9.1 Spesolimab and Imsidolimab

Both spesolimab and imsidolimab are humanized monoclonal antibodies against the IL‐36 receptor and are approved for generalized pustular psoriasis [94]. Given the IL‐36 involvement in NS, it represents a potential novel approach. A randomized, double‐blind, placebo‐controlled study to evaluate the efficacy and safety of monthly spesolimab injections (NCT05856526) is ongoing now [95]. In addition, a case report is published of a 5‐year‐old girl with NS who improved with spesolimab [80].

### 2.10 IFN Inhibitor

#### 2.10.1 Anifrolumab

Anifrolumab is a monoclonal antibody that binds to IFNAR1, blocking the activity of all type I IFNs and approved for systemic lupus erythematosus [61, 96]. No clinical use has been reported in NS, although type I IFN signature has been shown in scaly erythroderma NS patients [13].

### 2.11 TSLP Inhibitor

#### 2.11.1 Tezepelumab

Tezepelumab is a human IgG2λ monoclonal antibody that specifically blocks TSLP and is approved for use as add‐on maintenance therapy for severe asthma in adults and children aged 12 years and older [62]. There have been no reports of the use in NS to date; although the importance of the KLK5‐PAR2‐TSLP axis has been suggested in the pathogenesis of NS [97].

### 2.12 IgE Inhibitor

#### 2.12.1 Omalizumab

Omalizumab is a recombinant, humanized IgG1 monoclonal antibody targeting IgE, approved for moderate‐to‐severe allergic asthma, CSU, chronic rhinosinusitis with nasal polyps, and food allergy [63, 98]. There are several case reports and small case series in NS, but most show no or limited response [29, 52, 69, 81, 82].

## 3 Small‐Molecule Therapies in Netherton Syndrome

### 3.1 JAK Inhibitors

#### 3.1.1 Tofacitinib

Tofacitinib is a JAK1/3 inhibitor that blocks IL‐2, IL‐4, IL‐6, IL‐15, IL‐21, IFN‐γ, and so forth, and is approved for the treatment of RA, PsA, polyarticular course juvenile idiopathic arthritis (pcJIA), and UC [64, 99]. A case report is published of a 3‐year‐old boy with NS who improved with tofacitinib [65].

#### 3.1.2 Baricitinib

Baricitinib is a JAK1/2 inhibitor which blocks IL‐3, IL‐5, IL‐6, IL‐12, IL‐13, IL‐23, IFN‐α, IFN‐β, IFN‐γ, and so forth, and is approved for the treatment of RA, AD, alopecia areata (AA), and COVID‐19 pneumonia [64, 66, 100]. A case report is published of dupilumab‐resistant scaly erythroderma in a 20‐year‐old Japanese woman with NS, which condition improved with baricitinib [33]. Baricitinib is approved for use in children with AD in Japan and the EU, but its use in children with NS has not yet been reported.

#### 3.1.3 Upadacitinib, Abrocitinib

Upadacitinib is a selective JAK1 inhibitor which blocks IL‐2, IL‐4, IL‐6, IL‐15, IL‐21, IFN‐γ, and so forth, and is approved for the treatment of RA, PsA, AD, AS, CD, and UC [64, 101]. There are several case reports of the use in NS, and the effectiveness of the treatment varies widely [32, 35, 83, 84]. Abrocitinib is also a JAK1 inhibitor which blocks IL‐4, IL‐13, IL‐22, IL‐31, and so forth, and is approved for the treatment of AD [102]. Two case reports of the use in NS who improved are published [40, 85].

#### 3.1.4 Ritlecitinib

Ritlecitinib selectively inhibits JAK3 and TEC kinase family, and blocks IL‐2, IL‐4, IL‐7, IL‐9, IL‐15, IL‐21, and so forth, and cytolytic activity of T cells. It is approved for the treatment of AA [103]. There have been no reports of the use in NS to date.

### 3.2 TYK2 Inhibitor

#### 3.2.1 Deucravacitinib

Deucravacitinib selectively inhibits the tyrosine kinase 2 (Tyk2) protein, and blocks IL‐12, IL‐23, IFN‐α, and so forth [104]. This inhibitor is approved for the treatment of PP [104]. There have been no reports of the use in NS to date.

### 3.3 PDE4 Inhibitor

#### 3.3.1 Apremilast

Apremilast is a selective phosphodiesterase 4 (PDE4) inhibitor which suppresses immune cell activity, reducing the production of CXCL9, CXCL10, IFN‐γ, TNF‐α, IL‐2, IL‐8, IL‐12, IL‐23, and so forth [105]. This inhibitor is approved for the treatment of PP, PsA, BD, and palmoplantar pustulosis (PPP) [105, 106]. There have been no reports of the use in NS to date.

## 4 Conclusion and Future Perspectives

There are no formally approved targeted therapies in Netherton syndrome (NS), but an increasing number of case reports support the off‐label use of biologics and small‐molecule therapies, especially those targeting IL‐4, IL‐13, IL‐17, IL‐36, and JAK signaling pathways. Dupilumab and secukinumab are among the most frequently reported agents, often showing significant clinical benefit in some patients.

However, treatment responses are diverse, likely reflecting the underlying immunological heterogeneity. Recent multi‐omics analyses have revealed distinct immune profiles among NS subtypes, including Th2‐dominant, Th17/IL‐36‐dominant, and type I interferon‐inducible. These findings highlight the importance of personalized approaches based on cytokine profiling, transcriptomics, or clinical phenotype.

Current evidence is mainly limited to case series; international collaborative registries and randomized controlled trials are needed to collect long‐term data on efficacy, safety, and disease progression. These efforts are essential to establish consensus treatment guidelines for this rare and diverse disease.

In addition to immunomodulatory therapies, novel therapeutic strategies are being explored, including gene therapy, mRNA‐based approaches, and local or systemic protease inhibitors aimed at restoring LEKTI function. Advances in genome editing technologies such as CRISPR/Cas9 may eventually provide therapeutic options by directly targeting pathogenic SPINK5 mutations.

In the interim, cytokine‐targeted therapies will be a promising bridge between symptomatic treatment and future disease‐modifying interventions. Careful risk–benefit assessment is essential, especially in pediatric patients and those with severe comorbidities. Ultimately, multidisciplinary international efforts will be key to translating molecular biology insights into improved clinical outcomes for NS patients.

## Conflicts of Interest

S.M. has received research funding from Sun Pharma Co. Ltd. and Maruho Co. Ltd., and honoraria from Eli Lilly Japan K.K., UCB Japan Co. Ltd., AbbVie GK, Pfizer Japan Inc., Torii Pharmaceutical Co. Ltd., Sanofi, K.K., Boehringer Ingelheim Co. Ltd., and Maruho Co. Ltd. The remaining authors state no conflicts of interest. S.M. is an Editorial Board member of Journal of Dermatology and a co‐author of this article. To minimize bias, they were excluded from all editorial decision‐making related to the acceptance of this article for publication.

## Data Availability

Data sharing not applicable to this article as no datasets were generated or analyzed during the current study.
